# High‐resolution mass spectrometric analysis of myo‐inositol hexakisphosphate using electrospray ionisation Orbitrap

**DOI:** 10.1002/rcm.7935

**Published:** 2017-09-17

**Authors:** Catherine A. McIntyre, Christopher J. Arthur, Richard P. Evershed

**Affiliations:** ^1^ Organic Geochemistry Unit, School of Chemistry University of Bristol Cantock's Close Bristol BS8 1TS UK; ^2^ School of Chemistry University of Bristol Cantock's Close Bristol BS8 1TS UK

## Abstract

**Rationale:**

The phosphorus storage compound in grains, phytic acid, or myo‐inositol hexakisphosphate (IP6), is important for nutrition and human health, and is reportedly the most abundant organic phosphorus compound in soils. Methods for its determination have traditionally relied on complexation with iron and precipitation, acid digestion and measurement of phosphate concentration, or ^31^P NMR spectroscopy. Direct determination of phytic acid (and its homologues) using mass spectrometry has, as yet, found limited application to environmental or other complex matrices. The behaviour of phytic acid in electrospray ionisation high‐resolution mass spectrometry (ESI‐HRMS) and its fragmentation, both in‐source and via collision‐induced dissociation, have not been studied so far.

**Methods:**

The negative ion mass spectrometry and tandem mass spectrometry (MS/MS) of IP6, and the lower inositol pentakisphosphate (IP5), using an ESI‐Orbitrap mass spectrometer is described. The purity of the compounds was investigated using anion‐exchange chromatography.

**Results:**

IP6 is highly anionic, forming multiply charged ions and sodium adduct ions, which readily undergo dissociation in the ESI source. MS/MS analysis of the phytic acid [M−2H]^2−^ ion and fragment ions and comparison with the full MS of the IP5 reference standard, and the MS/MS spectrum of the pentakisphosphate [M−2H]^2−^ ion, confirm the fragmentation pattern of inositol phosphates in ESI. Further evidence for dissociation in the ion source is shown by the effect of increasing the source voltage on the mass spectrum of phytic acid.

**Conclusions:**

The ESI‐HRMS of inositol phosphates is unusual and highly characteristic. The study of the full mass spectrum of IP6 in ESI‐HRMS mode indicates the detection of the compound in environmental matrices using this technique is preferable to the use of multiple reaction monitoring (MRM).

## 1 INTRODUCTION

**Figure 1:**
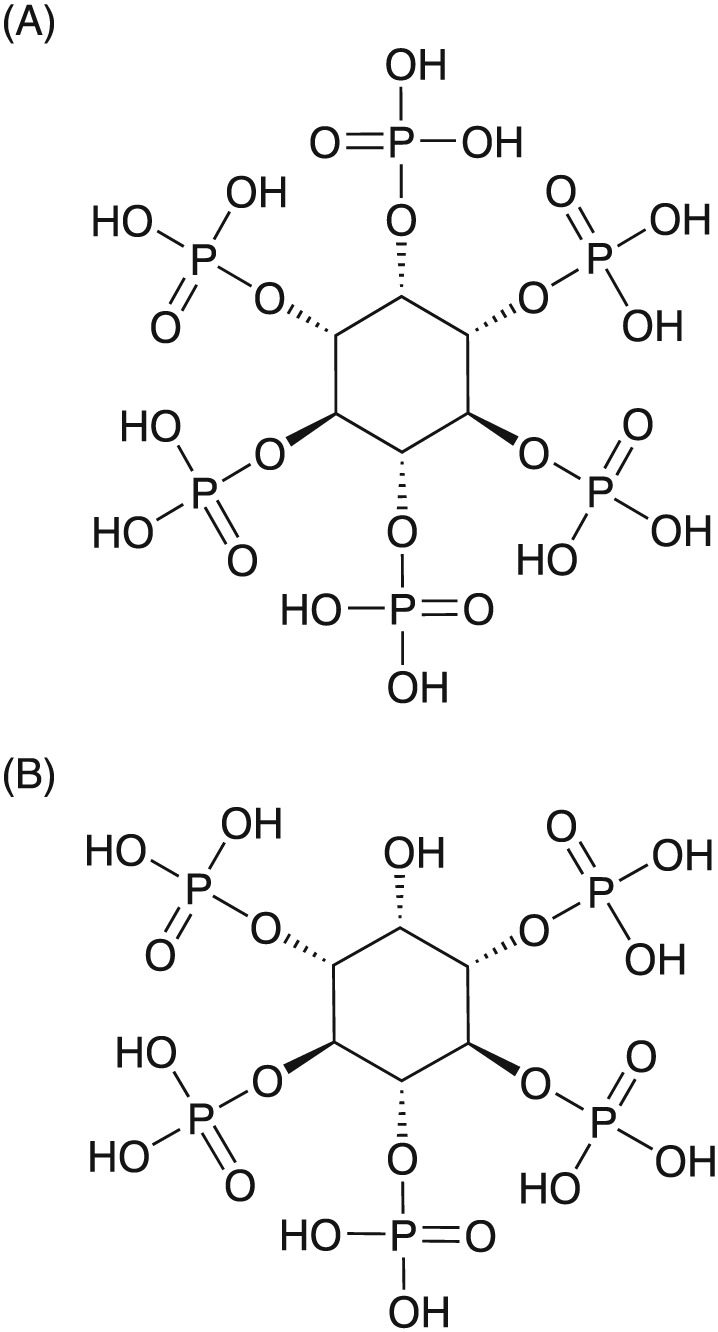
A, The structures of D‐myo‐inositol 1,2,3,4,5,6‐hexakisphosphate (IP6) and B, D‐myo‐inositol 1,3,4,5,6‐pentakisphosphate (IP5) as determined by Johnson and Tate.^4^ Monoisotopic masses are 659.8614 Da and 579.8950 Da, respectively

Organic phosphorus (P) can contribute to up to 80% of soil P, with implications for the availability of soil P to plants. Of this fraction, IP6 is reportedly the most abundant organic P compound in soils and sediments.^1^, ^2^, ^3^ IP6 (Figure 1A) is an unusual compound comprising an inositol ring with six bulky and very polar phosphate substituents. A series of homologous, lower inositol phosphates, including myo‐inositol pentakisphosphate (Figure 1B), is also found in nature.

Due to its importance, IP6 has been widely studied. It was first extracted in 1895,^4^ with studies in the following decades attempting to determine its chemical formula.^5^ Despite the structure of phytic acid not being confirmed until 1969 by ^31^P NMR,^6^ and in 1971 by X‐ray crystallography,^7^ it has been extensively studied and shown to be present in high concentrations in grains, seeds and plant roots. Due to its high abundance in soils, IP6 potentially plays an important role in the P biogeochemical cycle and indeed in the P enrichment of water bodies *via* transport from soils and sediments.

Initial determinations of IP6 began with acid extraction from seeds, followed by precipitation of IP6 with Fe(III) and measurement of the depleted concentration of Fe(III) in solution, thereby inferring the concentration of IP6.^8^ In 1977 Harland and Oberleas^9^ demonstrated the hydrolysis of IP6 using concentrated H_2_SO_4_ and HNO_3_ and the quantification of the released phosphate using the molybdenum blue test.^10^ The determination of IP6 along with the inositol phosphate stereoisomers from soils by Cosgrove^11^ in the 1960s was achieved by the hydrolysis of the inositol phosphates followed by paper chromatography of the inositol core. An alternative method for the determination of IP6 using phytase enzymatic digestion has also been used widely;^12^ the concentration of phosphate released from the digested IP6 is measured using molybdenum colorimetry. Phytases may, however, not be IP6‐specific, and may digest other phosphate‐containing compounds co‐occurring in complex environmental matrices.

In recent decades, a range of more instrumental analytical methods for determining IP6 has been developed. Liquid and anion‐exchange chromatography have been used to separate, identify and quantify inositol phosphates in food and biological samples on the basis of retention times and peaks areas.^13^, ^14^ The methods have contended with the presence of the homologous compounds, the lower inositol phosphates, e.g. pentakisphosphate, tetrakisphosphate, etc., and the stereoisomers of the inositol phosphates in the chiro, scillo, neo, etc., forms making chromatographic separation of the compounds difficult. These lower myo‐inositol phosphates are intermediates in the biosynthesis of IP6, and so are commonly found associated with IP6 in plant extracts. In ion‐exchange chromatography systems, IP6 detection uses electrochemical conductivity detection,^15^ or post‐column derivatisation with Fe(NO_3_)_3_ for spectrophotometric detection.^16^ Liquid chromatographic systems have also used refractive index detection of IP6,^17^ or more recently inductively coupled plasma mass spectrometry.^18^ Surprisingly few studies (see below) have employed direct determination of IP6 using mass spectrometry, perhaps because ion‐exchange chromatography liquid chromatography (LC) systems are generally incompatible with mass spectrometers due to metal components in the interface pumping systems and the high ionic strength of mobile phases.

Currently, ^31^P NMR spectroscopy is the principal method of characterisation of P in matrices such as soils and manures.^19^, ^20^, ^21^ This method is, however, compromised by the low sensitivity of NMR. Furthermore, the complexity of soil extracts results in multiple overlapping resonances in the diagnostic regions of the NMR spectrum. This makes unequivocal identification of individual compounds difficult,^22^ particularly if their relative concentrations are low. Identifications by ^31^P NMR spectroscopy in soil extracts usually rests on comparisons of resonances with literature values,^20^ or spiking experiments.^23^ In the absence of knowledge of the numbers and abundances of compounds contributing to an NMR spectrum, it is conceivable that the peaks identified as correlating to IP6 may derive from a number of unknown compounds which happen to have similar chemical shifts to IP6. The specificity of this method is therefore open to debate.

To date, there has been little work on the mass spectrometric analysis of IP6. One of the features of electrospray ionisation (ESI) is the formation of salt adducts with ions present in solution. These salt adducts can result in multiple analyte‐adduct ions, complicating the recorded spectra and reducing ion yields. This is particularly relevant in the case of IP6 where there is potential for the compound to form adducts with up to twelve cations. The complexity this adds to the identification of IP6 using ESI‐MS is seen in the report of Heighton et al^24^ where cations were added to the IP6 solution in order to use the formation of adducts to identify acid dissociation constants. Up to 16 ions are identified as IP6 per cluster in the spectrum with Fe^3+^, Na^+^ or Cu^2+^ adducts, or a mixture of these metals. The addition of different metals complicated, rather than aided, the interpretation of the mass spectra. Rougemont et al^25^ developed a method where ion‐pairing chromatography was used to separate IP6 from a whole blood matrix. The addition of modifiers to the LC eluent resulted in fewer adducts, and therefore simplified the mass spectra and improved the identification of IP6. Accurate mass analysis was, however, not employed in this study, nor was the behaviour of IP6 under ESI conditions studied.

Two studies^26^, ^27^ have aimed to determine inositol phosphates in sediments using multiple reaction monitoring (MRM) mass spectrometry. Identification of, not only IP6, but also the lower inositol phosphates (IP5, IP4, IP3, etc.), was on the basis of fragmentation reactions. A third study^28^ determined IP6 in wetland soils *via* size‐exclusion chromatography coupled to negative ion ESI‐MS. Here, identification of the elution of IP6 was on the basis of a selected ion mass chromatogram for *m/z* 659. These studies are complicated by the potentially labile elimination of metaphosphoric acid (HPO_3_) from IP6 resulting in fragment ions which are isobaric with lower inositol phosphate ions, a phenomenon identified by Cooper et al.^29^ Therefore, MRM may give false positives where fragment ions of IP6 are erroneously identified as lower inositol phosphates, and quantification on the basis of selected ion mass chromatography may be inaccurate.

This paper explores the mass spectrometry of IP6 using an ESI‐Orbitrap mass spectrometer and identifies the behaviour of IP6 in an ESI source. The adoption of multiple charges, formation of salt adducts and fragmentation pattern of the compound are identified. The MS and MS/MS behaviour of a lower inositol homologue standard, IP5, was also investigated in order to verify the fragmentation behaviour of IP6.

## 2 EXPERIMENTAL

### 2.1 Standard compounds

Reference standards IP6 (D‐myo‐inositol 1,2,3,4,5,6‐hexakisphosphate sodium salt, Na_12_C_6_H_12_O_6_(HPO_3_)_6_) and IP5 (D‐myo‐inositol 1,3,4,5,6‐pentakisphosphate pentapotassium salt, K_5_C_6_H_12_O_6_(HPO_3_)_5_) were purchased from Santa Cruz Biotechnology (Dallas, TX, USA). Solutions (20 ppm for MS, 10 ppm for qualitative IC, 130 ppm for preparative IC) were prepared with double distilled water.

### 2.2 Ion chromatography

A Dionex ICS‐5000 ion chromatograph (Thermo Scientific, Hemel Hempstead, UK) equipped with a KOH eluent generator, ion suppressor and conductivity detector was used for chromatographic separation, identification and quantification of reference standards IP6 and IP5. The compounds were separated using an Ionpac AS11 column (2 × 250 mm; Thermo Scientific) with an AS11G guard column (2 × 50 mm). The flow rate was set to 0.25 ml.min^−1^, and the column temperature to 30 °C. The elution gradient included a 10 min equilibration at 4 mM KOH, followed by: 0 min: 4 mM KOH, 19 to 24 min: 70 mM KOH, 29 to 30 min: 4 mM KOH. Eluate fractions were collected post‐detection at 30 s intervals in glass vials for HRMS analysis. Chromatograms were analysed in Chromeleon (Thermo Scientific).

### 2.3 High‐resolution mass spectrometry

MS analysis of IP6 and IP5 was performed on an Orbitrap Elite mass spectrometer (Thermo Scientific) with an ESI source. The Orbitrap was operated in negative ion mode, calibrated using negative ion calibration solution (Thermo Scientific) and tuned automatically on the *m/z* 328.9 (IP6 [M−2H]^2−^) ion. Solutions were directly infused at 10 μL.min^−1^ for acquisition of initial full mass spectra and at 6 μL.min^−1^ for MS/MS analysis. The source voltage was set to −1.8 kV, sheath gas (nitrogen) flow rate to 30 arbitrary units (arb), the auxiliary gas (nitrogen) flow rate to 0 arb and the sweep gas (nitrogen) flow rate to 1 arb. The capillary temperature was optimised at 275 °C. Full mass spectra were recorded at 120,000 resolution and 50 scans were averaged in order to increase the signal‐to‐noise ratio. MS/MS spectra were recorded at 15,000 resolution in order to allow a higher scan rate which would be useful for future LC/MS. MS/MS data was collected for the most abundant 20 ions in a spectrum. Fragmentation was *via* higher energy collisional dissociation (HCD) at 65% normalised energy. Mass spectra were analysed using Xcalibur (Thermo Scientific).

The effect of varying the source voltage was studied by maintaining the sheath gas flow rate at 30 arb, the auxiliary gas flow rate at 0 arb, and the sweep gas flow rate at 1 arb, and changing the source voltage from −1.0 kV to −3.6 kV in 0.2 kV increments.

IC fraction solutions were directly infused at 10 μL.min^−1^. The source voltage was set to −3.4 kV, the sheath gas flow rate to 30 arb, the auxiliary gas flow rate to 15 arb and the sweep gas flow rate to 9 arb.

## 3 RESULTS AND DISCUSSION

**Figure 2:**
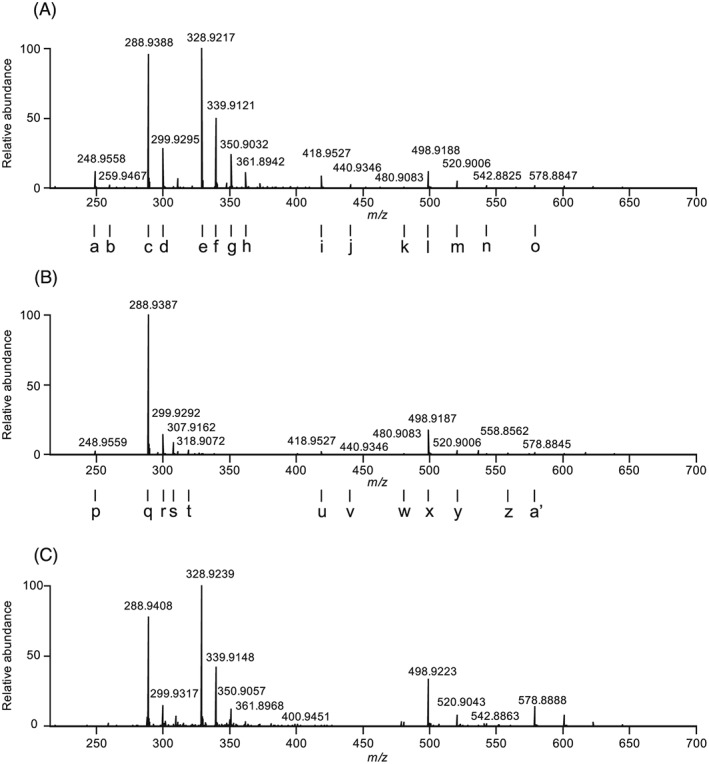
Negative ion mass spectra obtained by direct infusion on an ESI‐Orbtirap: A, IP6 reference standard, B, IP5 reference standard, and C, isolated IP6 in fraction 1 (F1, Figure 4b). Ions a to a' are detailed in Table 1

The HRMS negative ion mass spectrum of IP6 is presented in Figure 2A. The major ions in the mass spectrum are given in Table 1. The range of ions appearing in the mass spectrum include singly (i, j, k, l, m, n, o) and doubly charged (a, b, c, d, e, f, g, h) species. Intact IP6 is observed primarily as its doubly charged ion, [M−2H]^2−^ (e, *m/z* 328.9217), with no singly charged [M−H]^−^ ion appearing in the spectrum (theoretical *m/z* 658.8535). Sodium adducts of the doubly charged [M−2H]^2−^ ion are also observed (f–h).

**Table 1:** Ions, charge, formula and mass accuracy (ppm) in the full mass spectra of IP6 and IP5 (Figures 2a and 2b, respectively). The RHS column indicates the precursor ions giving rise to the product ions deduced by MS/MS

	Ion	z	Formula	ppm	Precursor ion (MS/MS)
	**IP6 spectrum (Figure** 2 **A)**				
a^a^	[M−2HPO_3_−2H]^2−^	2	C_6_H_14_O_18_P_4_	1.5	
b	[M‐2HPO_3_‐3H+Na]^2‐^	2	C_6_H_13_O_18_P_4_Na	1.5	
c	[M‐HPO_3_‐2H]^2‐^	2	C_6_H_15_O_21_P_5_	1.5	
d	[M‐HPO_3_‐3H + Na]^2‐^	2	C_6_H_14_O_21_P_5_Na	1.9	
e	[M‐2H]^2‐^	2	C_6_H_16_O_24_P_6_	1.8	
f	[M‐3H+Na]^2‐^	2	C_6_H_15_O_24_P_6_Na	2.5	
g	[M‐4H+2Na]^2‐^	2	C_6_H_14_O_24_P_6_Na_2_	2.3	
h	[M‐5H+3Na]^2‐^	2	C_6_H_13_O_24_P_6_Na_3_	2.2	
i	[M−3HPO_3_−H]^−^	1	C_6_H_14_O_15_P_3_	4.4	248.96, 288.94, 328.92,498.92
j	[M−3HPO_3_−2H+Na]^−^	1	C_6_H_13_O_15_P_3_Na	4.3	299.93
k	[M−2HPO_3_−H_2_O−H]^−^	1	C_6_H_13_O_17_P_4_	4.2	288.94
l	[M−2HPO_3_−H]^−^	1	C_6_H_15_O_18_P_4_	4.2	288.94, 328.92
m	[M−2HPO_3_−2H+Na]^−^	1	C_6_H_14_O_18_P_4_Na	4.3	299.93
n	[M−2HPO_3_‐3H+2Na]^−^	1	C_6_H_13_O_18_P_4_Na_2_	4.2	
o	[M−HPO_3_−H]^−^	1	C_6_H_16_O_21_P_5_	4.3	328.92
	**IP5 spectrum (Figure** 2 **B)**				
p	[M−HPO_3_−2H]^2−^	2	C_6_H_14_O_18_P_4_	1.3	
q	[M−2H]^2−^	2	C_6_H_15_O_21_P_5_	1.7	
r	[M−3H+Na]^2−^	2	C_6_H_14_O_21_P_5_Na	2.4	
s	[M−3H+K]^2−^	2	C_6_H_14_O_21_P_5_K	2.3	
t	[M−4H+NaK]^2−^	2	C_6_H_13_O_21_P_5_K	2.2	
u	[M−2HPO_3_−H]^−^	1	C_6_H_14_O_15_P_3_	4.4	
v	[M−2HPO_3_−2H+Na]^−^	1	C_6_H_13_O_15_P_3_Na	4.3	248.96, 288.94, 498.92
w	[M−HPO_3_−H_2_O‐H]^−^	1	C_6_H_13_O_17_P_4_	4.2	
x	[M−HPO_3_−H]^−^	1	C_6_H_15_O_18_P_4_	4.4	288.94
y	[M−HPO_3_−2H+Na]^−^	1	C_6_H_14_O_18_P_4_Na	4.3	288.94
z	[M−HPO_3_−2H+Na+K]^−^	1	C_6_H_13_O_18_P_4_NaK	4.5	299.93
a'	[M−H]^−^	1	C_6_H_15_O_21_P_5_	4.7	

a Letters correspond to annotated ions in the mass spectra shown in Figure 2.

The HRMS negative ion mass spectrum of the IP5 reference standard is given in Figure 2B. The major ions (Table 1) include the doubly charged ion, [M−2H]^2−^ (q, *m/z* 288.9387), and its sodium and potassium adducts (r, s, t).

**Figure 3:**
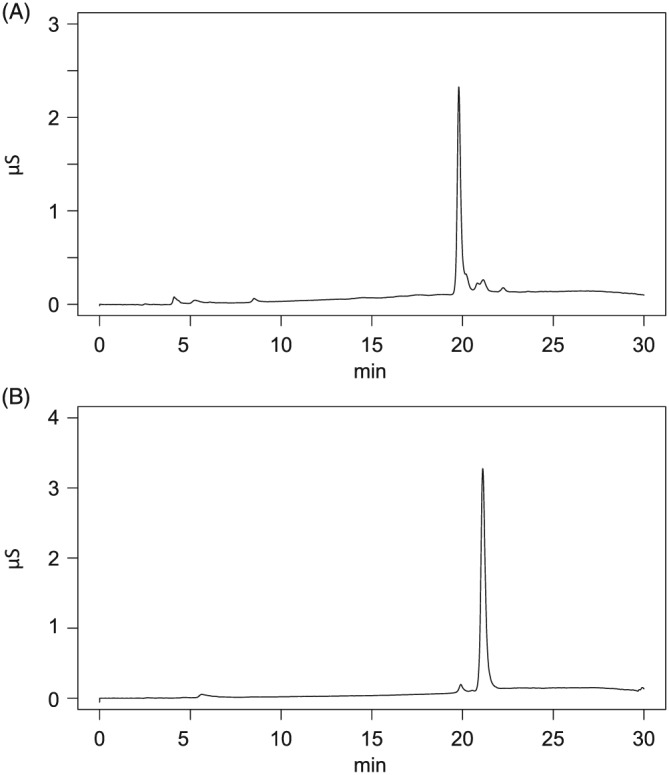
Ion chromatograms of A, IP6 and B, IP5 reference standards (10 ppm) obtained on a Dionex ICS‐5000 with an Ionpac AG11 column and KOH eluent. The IP6 standard is ~84% pure, while the IP5 standard is ~97% pure with IP6 contamination at ~20 min

Ions corresponding to the chemical formulae IP6 – xHPO_3_ –yH (a, c, i, l, o, Figure 2A) and IP5 – xHPO_3_ –yH (p, u, x, Figure 2B) are observed in both mass spectra. These ions are isobaric with lower inositol phosphate (IP5, IP4, IP3) ions. Therefore, lower inositol phosphate impurities in the reference standards may account for the presence of these ions in the mass spectra. The ion chromatograms (Figure 3) of the reference standards indicate that the IP6 standard is ~84% pure, and the IP5 standard is ~97% pure. Alternatively, the lower inositol phosphate ions may be formed through the loss of phosphate due to in‐source fragmentation of the IP6 ion, possibly *via* a 1,3‐hydride shift previously reported by Palumbo et al.^30^ The observation of ions corresponding to the loss of water and phosphate (k, w, Figure 2) also potentially indicates in‐source fragmentation.

The implications of in‐source fragmentation of IP6 under ESI conditions are relevant where MRM or selected ion mass chromatography is the method of analysis of inositol phosphates. If the compound fragments to give ions isobaric with lower inositol phosphate ions, the validity of the identification of these compounds would be called into question.

**Figure 4:**
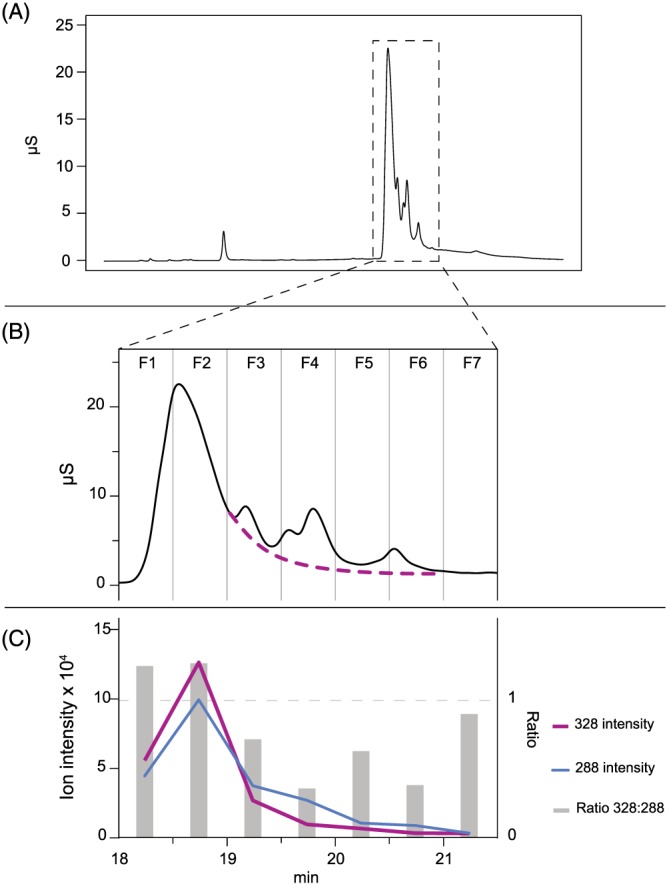
A, full ion chromatogram of 130 ppm IP6 reference standard, with enlarged region 18–21.5 min and fractions delineated in B. Fractions were collected from the IC system in 30 s intervals. Mass spectra of each fraction were then obtained by direct infusion to the Orbitrap in negative ion mode. C, corresponding ion intensities for *m/z* 328.92 (IP6) and *m/z* 288.94 (IP5 or IP6 [M−HPO_3_−2H]^2−^) in each fraction, along with the ratio of those ions. The IP6 peak is extrapolated (dotted line) in B, on the basis of the presence of the *m/z* 328.92 ion in the mass spectra [Color figure can be viewed at wileyonlinelibrary.com]

We therefore sought to confirm or refute the in‐source fragmentation hypothesis by purifying the reference standard. This was achieved by collecting fractions from the ion chromatograph. The mass spectrum of the leading front edge of the IP6 peak (the purest IP6 fraction F1, Figure 4B) is given in Figure 2C, and is very similar to that of the IP6 reference standard, confirming that the ions isobaric with IP5 and IP4 ions observed in the reference spectra arise from in‐source fragmentation of IP6 and not contamination by these homologues.

Further analysis of the mass spectra corresponding to the 30‐s fractions collected from the chromatograph, suggests that the IP6 peak tails into the later eluting peaks. IP6 appears in each fraction collected although its abundance reduces from fraction 3 to fraction 7. The IP5 [M−2H]^2−^ ion is the major ion in the mass spectrum for fractions 3, 4, 5, 6, and 7. The ratio of the ions *m/z* 328:288 in the mass spectra is constant for fractions 1 and 2 where the IP6 peak elutes. In the following fractions the ratio of *m/z* 328:288 falls below 1, as IP5 comes to dominate the spectra. This indicates that the minor peaks in the chromatogram are isomers of IP5, and that the IP6 peak elutes in fractions 1 and 2 and then tails through the chromatogram to fraction 7. The larger peak in fraction 4 was determined to be IP5, as confirmed by co‐injection of the IP6 and IP5 reference standards.

**Figure 5:**
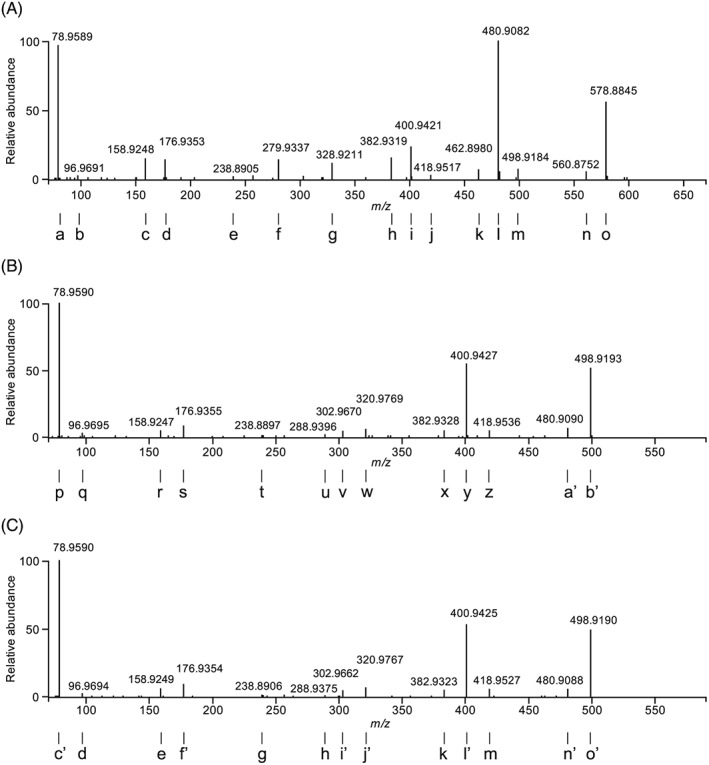
Fragmentation MS/MS HCD 65% NCE scans of precursor ions: A, *m/z* 328.92 (IP6 standard), B, *m/z* 288.94 (IP6 standard), and C, *m/z* 288.94 (IP5 standard). Ions a–o' are detailed in Table 2

MS/MS experiments were performed to study the fragmentation pattern of the IP6 and IP5 [M−2H]^2−^ ions under HCD conditions. The MS/MS spectra for both are given in Figure 5 and the ions identified in Table 2. The MS/MS spectrum of the *m/z* 288.94 ion from the IP6 reference standard is also presented. Analysis of the MS/MS fragmentation patterns of the precursor ions *m/z* 328.92 and 288.94 demonstrate that these ions fragment principally with the loss of HPO_3_. Loss of water from the precursor ion is more prevalent in these MS/MS spectra than in the full MS spectrum, indicative of the higher energy conditions in the HCD cell than in the ESI source. Precursor ions that give rise to product ions identified as fragments in the full MS spectrum are indicated in Table 1. Many of the ions have more than one precursor.

**Table 2:** Ions, charge, formula and mass accuracy (ppm) in the MS/MS product ion spectra of *m/z* 328.92 (IP6 standard), 288.94 (IP6 standard), and 288.94 (IP5 standard) (Figures 5A, 5B, and 5C, respectively)

	Ion	z	Formula	ppm
	**328.94 product ion spectrum**			
a^a^	Phosphate	1	PO_3_	−5.0
b	Phosphate	1	H_2_PO_4_	−0.3
c	Polyphosphate	1	HP_2_O_6_	0.2
d	Polyphosphate	1	H_3_P_2_O_7_	0.6
e	Polyphosphate	1	H_2_P_3_O_9_	2.8
f	[M−HPO_3_−H_2_O‐2H]^2−^	2	C_6_H_13_O_20_P_5_	1.3
g	[M−2H]^2−^	2	C_6_H_16_O_24_P_6_	2.7
h	[M−3HPO_3_−2H_2_O−H]^−^	1	C_6_H_10_O_13_P_3_	4.0
i	[M−3HPO_3_−H_2_O‐H]^−^	1	C_6_H_12_O_14_P_3_	4.7
j	[M−3HPO_3_−H]^−^	1	C_6_H_14_O_15_P_3_	6.8
k	[M−2HPO_3_−2H_2_O−H]^−^	1	C_6_H_11_O_16_P_4_	3.8
l	[M−2HPO_3_−H_2_O−H]^−^	1	C_6_H_13_O_17_P_4_	4.4
m	[M−2HPO_3_−H]^−^	1	C_6_H_15_O_18_P_4_	5.0
n	[M−HPO_3_−H_2_O−H]^−^	1	C_6_H_14_O_20_P_5_	2.6
o	[M−HPO_3_−H]^−^	1	C_6_H_16_O_21_P_5_	4.7
	**288.94 IP6 product ion spectrum**			
p	Phosphate	1	PO_3_	−6.3
q	Phosphate	1	H_2_PO_4_	−4.4
r	Polyphosphate	1	HP_2_O_6_	0.7
s	Polyphosphate	1	H_3_P_2_O_7_	−0.6
t	Polyphosphate	1	H_2_P_3_O_9_	6.1
u	[M−2H]^2−^	2	C_6_H_15_O_21_P_5_	0.2
v	[M−3HPO_3_−2H_2_O−H]^−^	1	C_6_H_9_O_10_P_2_	0.3
w	[M−3HPO_3_−H_2_O−H]^−^	1	C_6_H_11_O_11_P_2_	2.4
x	[M−2HPO_3_−2H_2_O−H]^−^	1	C_6_H_10_O_11_P_3_	1.6
y	[M−2HPO_3_−H_2_O−H]^−^	1	C_6_H_12_O_14_P_3_	3.2
z	[M−2HPO_3_−H]^−^	1	C_6_H_14_O_15_P_3_	2.3
a'	[M−HPO_3_−H_2_O−H]^−^	1	C_6_H_13_O_17_P_4_	2.7
b'	[M−HPO_3_−H]^−^	1	C_6_H_15_O_18_P_4_	3.2
	**288.94 IP5 product ion spectrum**			
c'	Phosphate	1	PO_3_	−6.3
d'	Phosphate	1	H_2_PO_4_	−3.4
e'	Polyphosphate	1	HP_2_O_6_	−0.4
f'	Polyphosphate	1	H_3_P_2_O_7_	0.0
g'	Polyphosphate	1	H_2_P_3_O_9_	2.4
h'	[M−2H]^2−^	2	C_6_H_15_O_21_P_5_	3.8
i'	[M−3HPO_3_−2H_2_O−H]^−^	1	C_6_H_9_O_10_P_2_	3.0
j'	[M−3HPO_3_−H_2_O−H]^−^	1	C_6_H_11_O_11_P_2_	3.0
k'	[M−2HPO_3_−2H_2_O−H]^−^	1	C_6_H_10_O_11_P_3_	2.9
l'	[M−2HPO_3_−H_2_O−H]^−^	1	C_6_H_12_O_14_P_3_	3.7
m'	[M−2HPO_3_−H]^−^	1	C_6_H_14_O_15_P_3_	4.4
n'	[M−HPO_3_−H_2_O−H]^−^	1	C_6_H_13_O_17_P_4_	3.2
o'	[M−HPO_3_−H]^−^	1	C_6_H_15_O_18_P_4_	3.8

a Letters correspond to annotated ions in the mass spectra shown in Figure 5.

**Figure 6:**
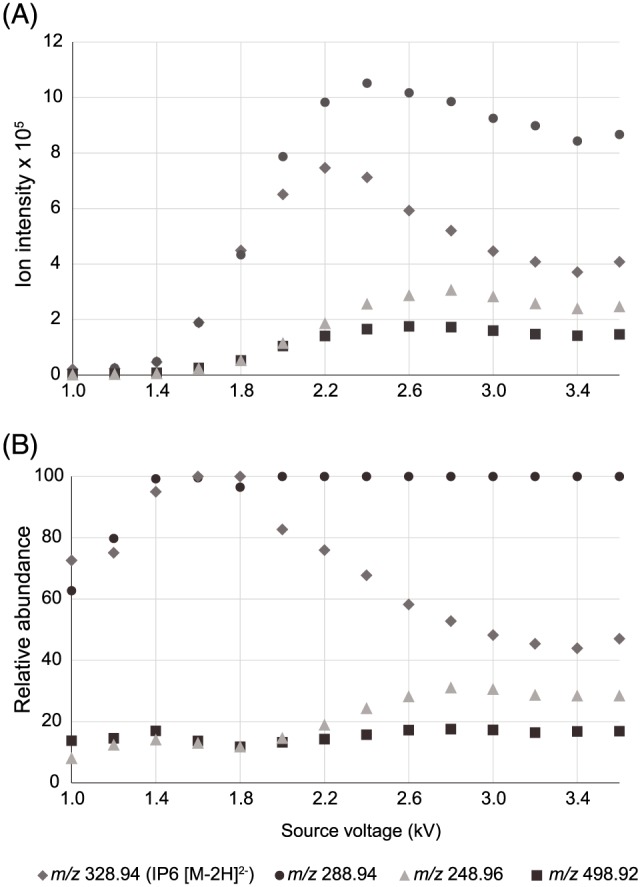
The effect of variation of source voltage in negative ion mode on an ESI‐Orbitrap Elite versus ion intensity A, and relative abundance B, of key ions in the mass spectrum of IP6 [Color figure can be viewed at wileyonlinelibrary.com]

The effect of changing the source voltage on the pattern of ions observed was determined and the results are presented in Figure 6. Varying the voltage from 1.0 to 1.4 kV revealed that the stability of the spray was poor with low ion intensity. The voltage was not raised above 3.6 kV as arc discharge in the source was observed at these voltages. Varying the voltage between 1.6 and 3.6 kV shows a clear trend emerge whereby the relative abundance of the doubly charged IP6 [M−2H]^2−^ ion decreases, while the relative abundances of *m/z* 288.94 and 248.96 increase, with *m/z* 288.94 becoming the dominant ion in the spectra. This again suggests that *m/z* 288.94 and 248.96 are fragments of the IP6 [M−2H]^2−^ ion and that increasing the voltage in the ESI source increases the extent of fragmentation of the compound.

Taken together, the mass spectra of the two reference standards, the data from the purified compound, the MS/MS data, and the effect of the source voltage on the mass spectrum of IP6, all demonstrate that the compound readily fragments in‐source under ESI conditions with the loss of HPO_3_ and water. The fragment ions are isobaric with ions from lower inositol phosphates and could therefore be mistaken for the presence of these compounds in a sample.

## 4 CONCLUSIONS

Negative ion electrospray Orbitrap mass spectra of IP6 and IP5 were recorded as part of a wider investigation aimed at incorporating the approach into a new analytical protocol for the assessment of the importance of inositol phosphates in environmental matrices. The investigation has revealed that:

1. The mass spectra of IP6 and IP5 are complicated yet offer a characteristic pattern of charge acquisition, fragmentation and formation of adducts of inositol phosphates in the ESI source.
2. The ion chromatographic assessment of the purity of the reference standards indicate that isobaric ions in the mass spectra are due to in‐source fragmentation and not lower homologue IP impurities.
3. The loss of water, as well as phosphate, and the fragmentation pattern seen in the MS/MS experiments, support conclusions regarding the mechanisms of fragmentation in the ESI source.
4. Analysis of the ion distribution with increasing source voltage provides further evidence of fragmentation.
5. Determination of inositol phosphates using ESI‐HRMS requires the study of the entire mass spectrum, as isobaric fragment ions can interfere with MRM experiments, giving false positive identification of lower inositol phosphates.
6. HRMS increases the certainty of identifications and is crucial for identification of inositol phosphates in complex matrices, such as plants and soil extracts.

The results of this investigation demonstrate the potential for using full scan ESI‐HRMS to study inositol phosphates, with clear gains to be made in incorporating the technique into protocols for the exploration of organic phosphorous cycling in the environment at the molecular level.
